# Towards a synthetic cell cycle

**DOI:** 10.1038/s41467-021-24772-8

**Published:** 2021-07-26

**Authors:** Lorenzo Olivi, Mareike Berger, Ramon N. P. Creyghton, Nicola De Franceschi, Cees Dekker, Bela M. Mulder, Nico J. Claassens, Pieter Rein ten Wolde, John van der Oost

**Affiliations:** 1 Laboratory of Microbiology, Wageningen University, Wageningen, The Netherlands; 2 Institute AMOLF, Amsterdam, The Netherlands; 3 Department of Bionanoscience, Kavli Institute of Nanoscience, Delft University of Technology, Delft, The Netherlands

**Keywords:** Synthetic biology, Cell division, Chromosome segregation, DNA replication

## Abstract

Recent developments in synthetic biology may bring the bottom-up generation of a synthetic cell within reach. A key feature of a living synthetic cell is a functional cell cycle, in which DNA replication and segregation as well as cell growth and division are well integrated. Here, we describe different approaches to recreate these processes in a synthetic cell, based on natural systems and/or synthetic alternatives. Although some individual machineries have recently been established, their integration and control in a synthetic cell cycle remain to be addressed. In this Perspective, we discuss potential paths towards an integrated synthetic cell cycle.

## Abstract (web summary)

A key feature of living cells is the cell cycle. In this Perspective, the authors explore attempts to recreate this process and what is still required for an integrated synthetic cell cycle.

## Introduction

Understanding the basic operating principles of life is a major scientific challenge, let alone using this knowledge to reconstruct a living cell from a basic set of essential parts. In recent years, significantly improved biological insights and impressive developments of molecular tools have been achieved. This prompted dreams to synthesise a living cell-like object by combining its molecular components. Attempts to create such ‘synthetic life’ will certainly contribute to addressing key fundamental questions on how life may have emerged and evolved, while elucidating its basic design principles. Furthermore, the generation of synthetic life forms may, on the longer term, provide novel platforms for a wide range of applications. A major breakthrough towards synthetic cells was the generation of a minimal cell by a top-down approach^1^. Using insights from transposon mutagenesis of a free-living bacterium with a small genome (1 Mb), *Mycoplasma mycoides*, a minimised functional genome was designed (530 kb). An innovative workflow including DNA synthesis, assembly of a complete circular chromosome, and its transplantation to a close relative of *M. mycoides*, eventually led to a minimal cell JCVI-syn3.0^1^. Recently, addition of a few genes related to cell division has restored the wild-type cell morphology and resulted in the creation of JCVI-syn3.0 + 126, consisting of 481 genes; remarkably, ~20% of these genes still have an unknown function^2^. Alternatively, several interdisciplinary teams are currently developing even more challenging bottom-up approaches^1^, aiming to combine well-studied molecular components of different origins to eventually construct a living synthetic cell^3,4^ (Table 1). These bottom-up approaches require the development of functional modules as well as their stepwise integration^5^.

**Table 1:** Synthetic cell modules.

Module	Potential solution	Ref.
Container	Liposomes, water-in-oil droplets, coacervates	^41,42,107^
Chromosomal configuration	Linear or circular, single/complete or multiple/partial chromosomes, single/multiple copies	^14,15,17^
Transcription and translation	In vitro transcription-translation systems (IVTT): PURE, TX-TL system	^4^
Energy	Arginine breakdown pathway, decarboxylase pathways, proton-pumping rhodopsins combined with ATP synthase	^3,113^
Cell growth	Lipid biosynthesis, vesicle fusion	^4,114–117^
DNA replication	ϕ29, T7, T4, *Escherichia coli* replicative machineries	^4^
DNA segregation	Random or active partitioning, entropy-driven segregation	^21,23,33^
Cell division		
Symmetry breaking	Reaction-diffusion, lipid phase separation, DNA partitioning	^33,43,48,49,51^
Membrane deformation	FtsZ, Cdv, actin-processing proteins, Min system, microtubules, lipids, DNA origami, mechanical deformation	^52,62–64,67,74,75^
Membrane abscission	ESCRT-III, dynamin, active droplets, mechanical splitting	^55,72–74^

Different modules and processes that are considered essential to create a synthetic cell and alternatives proposed in the literature.

**Fig. 1:**
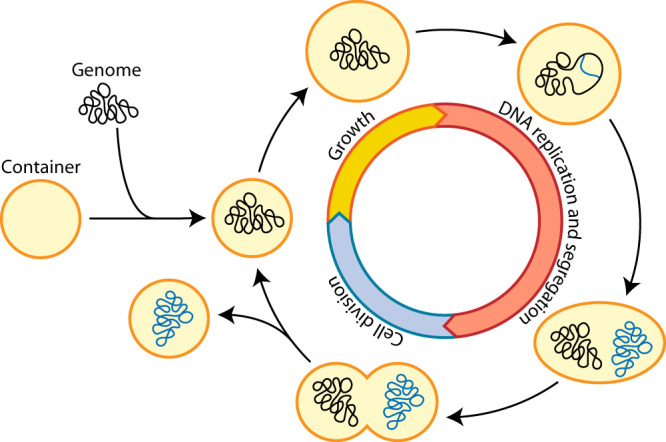
The natural and synthetic cell cycle. A natural/synthetic cell consists of a genome expressing all essential components inside a container. After a first phase of growth (yellow), the cell enters a phase in which the genomic content is replicated and segregated (red). Finally, cell division happens in the last phase of the cell cycle (blue), generating two daughter cells. Note that, although the first phase of the cell cycle is one of growth, the cell continuously grows also during DNA replication, DNA segregation and cell division.

There is no single definition of what constitutes a ‘living’ cell. Yet, arguably the defining characteristic of a living cell is its ability to make a copy of itself. In other words, living cells can grow and divide autonomously, which implies that during each cell cycle on average all its components are faithfully duplicated and partitioned over the daughter cells (Fig. 1). The modules involved in that process are DNA replication, DNA segregation, cell growth and cell division, which together form the cell cycle. In addition, the proteins that drive and control these processes need to be produced, which means that a minimal cell also should include a transcription-translation machinery. An early proposal suggested that this already requires at least 151 genes in a 113 kb genome^6^. Another key feature of all forms of life, including a synthetic cell, is that these processes co-occur in some form of ‘cellular’ compartment or container. Moreover, some basic metabolism is also required for providing the energy and facilitating the reducing power and building blocks for biosynthesis of all the crucial components (e.g. DNA, RNA, proteins, lipids, co-factors). At least to some extent, however, these building blocks may be supplied externally. Hence, we conclude that a minimal synthetic cell should at least contain a cell cycle that combines DNA replication and segregation with cell growth and division.

In this Perspective, we discuss major challenges and potential approaches to create a synthetic cell cycle. For comprehensive information about natural cell cycles, metabolic modules as well as general engineering and evolution strategies towards a synthetic cell, we refer to some excellent recent reviews^3–5^. Below, a comparison is made of promising modules for DNA replication, DNA segregation, and cell division. In addition, cell growth is obviously an essential feature of a living cell, and some examples are available of coupling the cell cycle with growth. However, in this perspective article we do not discuss in detail reconstitution of biosynthesis routes for lipids or other essential building blocks that are also at the basis of cellular growth. For an extensive overview of such processes, we thus refer to a comprehensive recent review^4^. As coordination of DNA replication, DNA segregation and cell division is considered crucial, they should be integrated with mechanisms that monitor cell volume (growth) and DNA content. Altogether, these modules and their control will provide a basis for achieving the grand scientific goal of a growing and stably dividing synthetic cell. After discussing potential promises and pitfalls of the different modules and mechanisms, we will provide an integrated outlook on what a synthetic cell cycle could look like.

## DNA replication: simple yet controlled

Every living cell executes DNA replication to ensure that its daughter cells will inherit a copy of the genomic content. Many efforts have been made to reconstitute this process extra-cellularly. An initial attempt to rebuild the DNA replication machinery of the model bacterium *Escherichia coli* set out to express the 13 core genes of its replication system in an in vitro transcription/translation system (IVTT)^7^. However, this minimal system did only result in synthesis of the complementary strand of a single-stranded circular DNA template^7^. For fully functional replication, including lagging strand synthesis and dissociation of the sister chromosomes, additional genes are required. Next, the full *E. coli* replication system (encoded by 25 genes) was reconstituted from purified proteins, resulting in one round of replication of circular double-stranded DNA templates up to 200 kb^8^. Production of a full *E. coli* replication system within IVTT reactions has not yet been reported, probably because of its relatively complex nature, the relatively large number of components, and the lack of chaperones in IVTT systems^7^.

**Fig. 2:**
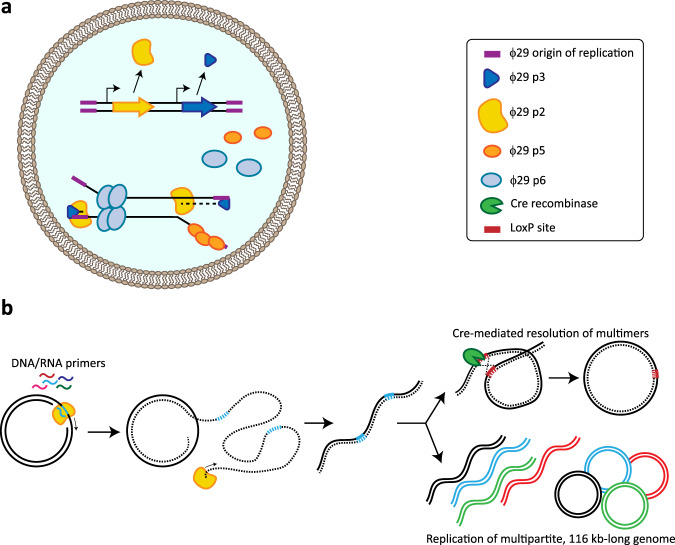
Synthetic DNA replication. Implementation of the bacteriophage ϕ29 system for DNA replication in in vitro transcription-translation (IVTT) reactions. **a** The reconstitution of the complete ϕ29 DNA replication machinery has been demonstrated inside a synthetic liposome. Bi-directional self-replication of a linear DNA has been achieved, with the linear DNA template encoding the DNA-polymerase (DNAP, protein p2) and the Terminal protein (p3), which binds to the origin of replication at the end of the linear fragment and provides an hydroxyl group to prime DNA-polymerisation. The single-stranded DNA-binding protein (p5) and the origin-binding protein (p6), promoting the unwinding of the phage origin of replication, were supplemented as purified proteins^14^. **b** ϕ29 DNAP-mediated rolling-circle amplification of circular dsDNA molecules, resulting in a linear dsDNA multimer^15^. The synthetic multimer can be re-converted into circular DNA monomers through Cre-Lox recombination^16^ (top). In a ϕ29 DNAP-based replication experiment using a multipartite genome^17^ (bottom right), spontaneous monomerisation, disentangling and circularisation was observed, notably resulting in a mixture of linear multimers and circular monomers^17^.

A promising, simpler alternative to achieve replication in synthetic cells is the single-protein DNA polymerase (DNAP) of bacteriophages. Several of these replication systems have been reconstituted in vitro^9,10^, with the machinery of bacteriophage ϕ29^11^ being investigated in most detail. The ϕ29 system consists of a DNAP and three associated proteins (Fig. 2a). Unlike other well-known phage DNAPs, ϕ29-DNAP exhibits strand-displacement activity that circumvents the need for additional helicases to unwind the upstream duplex DNA^12^. The ϕ29 DNAP has been demonstrated to achieve high replication rates of linear DNA fragments up to 70 kb in vitro^13^. Primer-independent initiation of replication from defined origins of replication by the ϕ29 DNAP requires the associated proteins. The use of the phage DNAP and associated proteins allowed for self-replication of linear DNA in IVTT reactions, where ϕ29 origins of replication were placed at both ends of the DNA fragment^14^. This strategy was also demonstrated to result in DNA replication within a liposome container (Fig. 2a)^14^. Compartmentalised self-replication is an essential step towards synthetic life, as well as a suitable approach for in vitro evolution of other modules^5^.

The ϕ29 DNAP has also been reported to mediate rolling-circle amplification of double-stranded circular DNA in IVTT mixtures. In the absence of the associated proteins, this DNAP can use either DNA^15^ or RNA^16^ oligo-nucleotide primers to generate long linear DNA multimers that need to be processed back to circular chromosomes before cellular division^15^. A follow-up study demonstrated splitting and re-circularisation of these multimers through Cre-Lox recombination^16^ (Fig. 2b). Moreover, it was shown that ϕ29 DNAP produced in IVTT reactions could amplify a set of 11 plasmids in parallel, covering a total size of 116 kb^17^ (Fig. 2b).

Even though ϕ29 DNA replication is currently the best developed system for eventually driving replication in a synthetic cell, there are relevant bottlenecks that need to be addressed. Firstly, the DNA amplification rates of the ϕ29 system in IVTT conditions should be optimised^17^, through adjusting the standard composition of IVTT reaction mixtures (PURE), and/or through laboratory evolution of the ϕ29 system itself^5,18^. Secondly, it is important to consider that the natural function of this phage system is to replicate a linear genome. This may imply restrictions for the actual genomic configuration of the synthetic cell, such as to use linear chromosomes, which could be a viable option, although most small genomes in cells (e.g. those in bacteria) are circular. Alternatively, the ϕ29 system could be adjusted to replicate circular chromosomes. For circularisation, the aforementioned Cre-Lox approach could be used, which was achieved by directed evolution of the initial DNA template^15^. Initial indications of spontaneous resolution of these multimers into circular chromosomes under IVTT reaction conditions^17^ should otherwise be investigated in more detail. Another bottleneck of the ϕ29 system may be that the processivity of its DNAP (responsible for replicating its 20 kb genome) is not sufficient to enable replication of a single chromosome of the size needed for a synthetic cell^13,14^. As splitting the genome into multiple smaller chromosomes is not preferred (see below; DNA segregation), a potential solution could be to improve the processivity of ϕ29 DNAP by laboratory evolution^5^. As another route, replicase systems from known viruses with larger, circular dsDNA genomes (250–500 kb) might be suitable candidates for replicating the genome of a synthetic cell.

In addition, an important limitation of viral replicative systems is the lack of regulation, as these systems evolved to drive uncontrolled amplification of viral DNA fragments. Appropriate timing of genomic replication, however, is essential for the fitness of biological cells^19^, and most likely for synthetic cells as well. To obtain a synthetic cell surviving throughout generations, we envision the introduction of control over replication initiation as an essential step, as discussed in more detail below.

## DNA segregation: using a biological or a physical approach

A cell does not only need to duplicate its genome, but it also needs to make sure that the generated sister chromosomes are properly partitioned over the daughter cells. This implies that, seemingly against the odds of entropy, a spatial order should be established through disentangling and separating the two chromosome copies. Well-known natural modules that drive this segregation process are the mitotic spindle apparatus of eukaryotic cells^20^ and the bacterial Par system^21^. The DNA segregation module of a synthetic cell could be adopted from the latter systems, but alternatively could also consist of a much more minimal mechanism. In order to be fully functional and controllable, the DNA segregation module should accomplish three main tasks: (i) breaking the symmetry to initiate disaggregation, (ii) achieving complete spatial segregation, and (iii) ensuring correct partitioning over the daughter cells. There are several alternative scenarios to achieve these tasks, each with consequences for the configuration of the chromosome, and for the type of DNA replication machinery of such a cell.

**Fig. 3:**
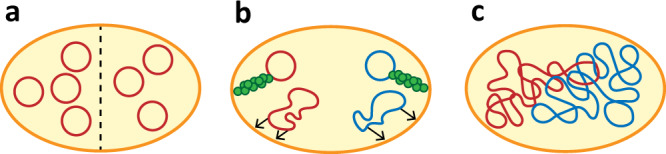
Synthetic DNA segregation mechanisms. **a** Random partitioning of DNA molecules, generating viable offspring in synthetic cells with sufficiently high genome copy numbers. **b** Molecular machineries for active partitioning of DNA molecules, such as the Par system (top) or membrane anchors in elongating cells (bottom). **c** Entropy-driven segregation, relying on the physical behaviour that large overlapping DNA macromolecules constrained in a small volume will spontaneously get separated from each other.

The simplest solution to accomplish appropriate segregation of identical genomes would be through random partitioning (Fig. 3a). This model was originally proposed for plasmid partitioning^22^, and relies on a large number of relatively small, identical DNA molecules. This sharpens the peak of the binomial distribution of inherited copies over the daughter cells to such an extent that the probability of a daughter cell receiving only a few or no copies of DNA becomes acceptably low. However, mathematical modelling of such a stochastic partitioning for other cytosolic macromolecules (e.g. proteins), shows that this results in major heterogeneity^23^. According to this model, random partitioning requires many copies of the chromosome, possibly in the order of tens. Replication of such a high number of DNA molecules would result in a huge burden, even when a designed synthetic cell may require a smaller genome (250–500 kb) than most bacteria. In addition, this approach would require precise fine-tuning of transcription and translation of all essential coding sequences, to avoid issues with imbalanced gene expression. Overall, we consider this solution inappropriate for DNA segregation in a synthetic cell.

Alternatively, an active molecular machinery can be implemented to achieve DNA segregation (Fig. 3B). Although a minimal mitotic spindle has successfully been reconstituted in vitro^24^, this system might still be too complex to be properly integrated and coordinated in a synthetic cell. Instead, inspiration can be taken from the bacterial world, in the form of plasmid or chromosomal segregation machineries. The bacterial actin-like plasmid partitioning (Par) system has been reported to push coupled plasmids apart^21^, or to space them regularly along the nucleoid^25^, contributing to equal partitioning. Regarding bacterial chromosomes, there is a long list of proposed mechanisms, ranging from dynamic anchoring to the poles or to other sites at the elongating membranes of growing cells^26,27^, to directional biases in either transcription or replication^28,29^. Yet, it is unlikely that any of these mechanisms alone would be sufficient to achieve full segregation^30^. Moreover, and especially relevant in the perspective of a synthetic cell, all these mechanisms require coordinated interactions between the involved structures, inevitably leading to an overwhelming complexity.

Because of the downsides of the previous options, it is interesting to explore a third scenario, namely entropy-driven segregation (Fig. 3c). This possibility relies on in silico observations that long polymer chains tend to segregate spontaneously when sufficiently spatially confined^31^. This effect can be explained by the strong increase in the entropic cost associated with overlapping polymers when they are constrained to volumes much smaller than their natural size^32^. There are at least three requirements for entropic segregation to occur. The primary one is a sufficient spatial confinement, and thus a minimal mass (size and density) of the polymers involved^33^. For instance, it is unlikely that entropic segregation could drive partitioning of a 500 kb genome in cells with a diameter of more than a few micrometres. If the totality of the coding sequences envisioned would not provide sufficient length, non-functional “filler” DNA could be incorporated in the genome. Secondly, the confinement shape plays a major role, which is why entropic segregation has been extensively explored for elongated cylindrical shapes like that of the *E. coli* cell^34^. For a synthetic cell that starts out spherical, the required symmetry breaking may be achieved by the constriction machinery needed for division (see below, Cell division). Lastly, entropy-driven segregation can be optimised by adapting the DNA topology, for example by introducing circularisation^35^, supercoiling or loop-creating cross-links^36^, which can be induced by recently established molecular mechanisms^37^. Furthermore, macromolecular crowding can increase the effective degree of confinement through the depletion effect^38^.

It is important to consider that the requirement of a relatively large DNA molecule would influence the choice of the replicative system. Another caution is that entropy-driven DNA segregation has so far been based on in silico predictions that are supported by indirect evidence only^39,40^. Hence, verification of entropy-driven partitioning of 250–500 kb genomes in different containers is required to decide on the suitability of this approach. Nevertheless, the strongest argument for entropy-driven segregation as the mechanism of choice for a synthetic cell is that its success does not hinge on precisely tuned biochemistry, but rather on a generic physical principle. In our perspective, this potentially makes it more robust, and therefore more likely to work independent of the constraints imposed by the other functional modules.

## Cell division: usual suspects and out-of-the-box alternatives

**Fig. 4:**
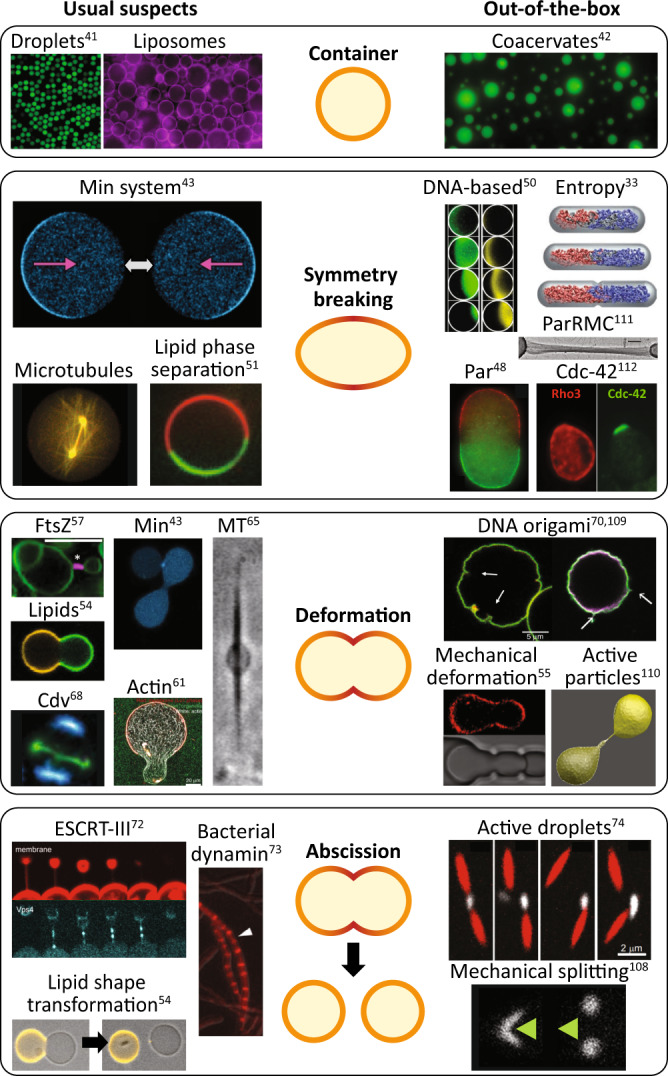
Synthetic cell division. Alternative synthetic cell container and the stages of symmetry breaking, deformation, and abscission during cell division. For each step, well-characterised (left) and alternative “out-of-the-box” candidates (right) are presented. Images depicting droplets, coacervates, lipids and lipid shape transformation, mechanical deformation, FtsZ, and DNA origami are reproduced from refs. ^41,42,54,55,57,70^, respectively, under the CC BY 4.0 licence. Images depicting Min system, Min are reproduced/adapted from ref. ^43^ under the CC BY-NC 4.0 licence. The image depicting MT was reproduced from ref. ^65^ (10.1021/acs.langmuir.6b00799) and further permissions related to the material excerpted should be directed to the ACS. The image depicting mechanical splitting was reproduced from ref. ^108^ (10.1021/acsnano.7b08411) and further permissions related to the material excerpted should be directed to the ACS. The image depicting DNA origami was reproduced from ref. ^109^ under the CC BY 3.0 licence. The image depicting Par is reproduced/adapted with permission from Development from ref. ^48^. Images depicting entropy symmetry breaking, actin, and active particles were reprinted by permission from refs. ^33,61,110^, respectively. The image depicting ParRMC is reproduced from ref. ^111^. Reprinted with permission from AAAS. The image depicting ESCRT-III is reproduced from ref. ^72^. Reprinted with permission from AAAS. The image depicting Cdv is reproduced from ref. ^68^. Reprinted with permission from AAAS. The image depicting DNA-based symmetry breaking was reprinted with permission from ref. ^50^; copyright 2013 American Chemical Society. Images depicting Cdc-42 were reproduced from ref. ^112^, with permission from American Society for Microbiology. Images depicting bacterial dynamin and active droplets were reproduced from refs. ^73,74^, respectively, with permission from Proceedings of the National Academy of Sciences of the United States of America. The image depicting lipid phase separation was reproduced from ref. ^51^, with permission from Europhysics Letters. The image depicting microtubules was kindly provided by Prof. Marileen Dogterom (TU Delft).

After chromosome segregation, cells proceed with the division into daughter cells. Conceptually, cell division is a multistep process that can be broken down into three distinct steps: symmetry breaking, membrane deformation, and membrane abscission. Regarding their implementation in molecular systems, one could adopt the well-studied natural cell division machineries, but also several “out-of-the-box” alternatives (Fig. 4).

Before considering division strategies, it is important to briefly consider the pros and cons of different types of synthetic containers. Water-in-oil droplets are an attractive option for testing purposes: very easy to mass-produce through microfluidics^41^ and offering excellent encapsulation of components. Yet, the large surface tension makes these droplets difficult to deform, and the external hydrophobic environment hardly allows any transport in and out of the synthetic cell. Another interesting container candidate is based on the recently rediscovered coacervates^42^. In this case liquid droplets are formed upon mixing two immiscible liquid phases, which would facilitate easy exchange of material due to the lack of a physical boundary. Coacervates, however, can spontaneously fuse, making it difficult to maintain a clear identity of protocells and hindering their use as containers for a synthetic cell. Finally, liposomes represent an excellent minimal model for a container of a synthetic cell. Their lipid bilayer mimics natural cell membranes, structures that can potentially be equipped with molecular machineries to deform and divide^43,44^. The non-permeable bilayer will prevent the influx of building blocks, but this can be overcome by the reconstitution of functional channels and transporters^45^. Based on these arguments and on the large amount of available knowledge, liposomes are to date the most obvious candidates as containers for a synthetic cell.

Cell division inevitably calls for symmetry breaking as a first step in the process of generating offspring. Several physical and biochemical mechanisms can be considered to induce asymmetries. Reaction-diffusion at the membrane can generate protein gradients and set up polarity. The best-studied example of reaction-diffusion is the Min system of rod-shaped bacteria like *E. coli*^43,46^, which uses pole-to-pole oscillations to define the division site at the cell midpoint. This Min system can also segregate membrane-bound proteins to spatial gradients along the cell length^47^. Although more complex, alternative reaction-diffusion systems are the metazoan PAR system^48^ (not to be confused with the aforementioned bacterial Par system) that produces a stable concentration gradient along the membrane, and the Cdc-42 system^49^ that localises the division site in budding yeast. Interestingly, fully synthetic DNA-based reaction-diffusion systems have also been demonstrated to create chemical waves in solutions in closed reactors^50^. Although still relatively complex, such systems may provide spatial cues for division and can be expanded to other rationally designed modules. Lipid phase separation is another well-characterised and robust process to break symmetry within a liposome^51^. However, here the downside is the requirement for an accurately controlled lipid composition throughout multiple generations in a synthetic cell. The systems involved in DNA segregation, discussed in the previous section, may also be of interest for synthetic cell division, since they break symmetry by partitioning sister chromosomes to the poles of the cell. For example, entropy-driven segregation^31,33^ in rod-shaped vesicles may help to identify a middle plane through nucleoid occlusion^52^. Nonetheless, for now reaction-diffusion systems seem to represent the most promising choice to achieve symmetry breaking as they have been very well characterised. The Min system is considered to be of particular interest, as pole-to-pole oscillations have already been established in liposomes^43^.

During the division process significant changes occur in the shape of the cellular container. In vivo, cell deformation is accompanied by excess membrane area that is generated by growth. In in vitro studies, this has often been achieved by applying an osmotic pressure difference across the membrane^53^. Osmotically deflated vesicles revealed that periodic Min protein binding can result in dumbbell-shaped liposomes^43^, which is an important step towards abscission, although completion of the cell division process has not been accomplished yet in vitro. In mixed-lipid liposomes, the line tension at the interface between two lipid phases can deform and even split the liposome, albeit typically asymmetrically^54^. External interventions in the form of mechanical deformation of the vesicles using microfluidic traps^55^ offer good spatial control, and may be very useful in initial stages of the synthetic cell research. However, for achieving a fully autonomous synthetic cell, this eventually needs to be substituted with a dedicated division machinery.

In natural cells, most deformations are controlled by dedicated protein machineries. By far the best-studied system of this type is the bacterial division protein FtsZ that orchestrates membrane constriction by a contracting Z-ring. This system is appealing due to its simplicity. Moreover, FtsZ rings were proven to localise to membrane necks in liposomes^56,57^. Interestingly, the Min system has been proven to be able to dynamically localise FtsZ near mid cell^58^. However, it is currently unclear whether in vitro the Z-ring can exert sufficient force to constrict the membrane of the synthetic cell to the point of division, or if instead its mere role is to recruit other proteins that actually drive this process. Recent studies reported that FtsZ is able to stabilise membrane deformations such as local buds^56,57,59,60^, but a dynamic constriction from large to small diameter rings remains to be demonstrated. Eukaryotic actin^61^ is another interesting filamentous protein that, in concert with actin-processing motor proteins, is able to induce blebbing^62^, protrusions and vesicle elongation^63^, as well as reversible spindle-like vesicle deformation^64^. These proteins can exert high forces on a membrane in a spatially controlled way, but their implementation seems less attractive due to the large number of components required. Alternatively, microtubules are a relatively simple cytoskeletal system able to deform membranes, either autonomously through polymerisation^65^ or by coupling to the motor protein kinesin^66,67^. However, microtubules generate a linearly extended rather than a dumbbell-shaped vesicle, hindering division. Another system that recently is receiving increasing attention is the archaeal Cdv system^68^. Cdv employs a constriction mechanism similar to the eukaryotic ESCRTs, its evolutionary homologue. While in vitro Cdv reconstruction remains to be established, the relative stability of these archaeal proteins may be an advantage when used in a bottom-up approach.

On a different note, DNA origami nanotechnology^69^ holds great potential to design functional components with tailored features such as local bending of the membrane^70,71^. However, implementation in a synthetic cell poses several challenges, such as achieving a proper folding of the origami or the need of an energy-burning module to achieve active constriction. On top of this, DNA origami cannot be synthesised in a cell. Some of these issues can, however, likely be tackled with the use either of RNA origami or of accessory DNA-binding proteins, but additional research is needed to demonstrate the feasibility and use of this solution in synthetic cells.

In the final stage of division, the membrane is fully pinched off and two separate daughter cells are born, ready to undergo a new cell cycle round. Membrane abscission is so far the least studied phenomenon when it comes to reconstituted systems. The eukaryotic ESCRT-III system has recently been reconstructed and proven to be functional in vitro^72^. This work, however, highlighted the challenges involved in controlling this protein complex both spatially and temporally. A simpler system is the bacterial dynamin that is recruited by the Z-ring^73^. Further investigation is needed to assess whether dynamin is sufficient to efficiently mediate abscission on its own. Due to its natural connection with FtsZ, the bacterial dynamin system is a promising candidate to accomplish membrane abscission and drive cell division for a synthetic cell. Regarding non-natural solutions, “active droplets” of coacervates with chemical reactions occurring inside have been shown to be able to autonomously split using proteins such as actin and myosin^74^. Assessing whether such coacervates can be encapsulated in vesicles and drive their division is another interesting future perspective^75^ that needs to be tackled, together with preventing spontaneous fusion between coacervate droplets.

## Cell cycle control

**Fig. 5:**
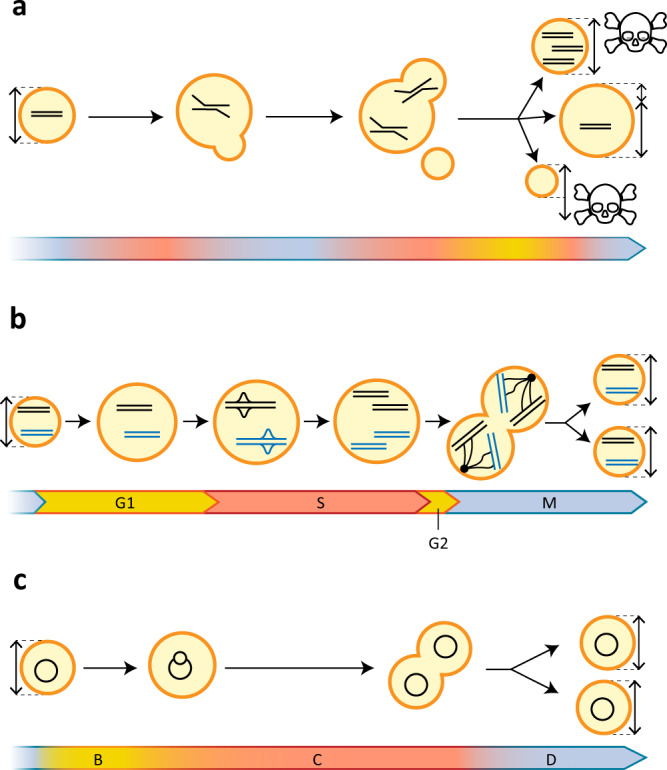
Cell cycle in natural and synthetic cells. **a** In a synthetic cell that lacks control over DNA replication, DNA segregation and cell division, nothing prevents these processes from happening simultaneously, most likely leading to loss of cell-size homoeostasis, hyper-replicative stress, inconsistency in genomic content, and non-viability of daughter cells. **b** In eukaryotic cells, the passage through phases of the cell cycle is controlled by molecular checkpoints, depicted as bold lines separating the different phases. **c** In the bacterium *Escherichia coli*, the cell cycle lacks a eukaryotic-type checkpoint system. However, the order of the cell cycle phases is maintained through coupling with growth.

Obtaining an autonomously replicating cell by bottom-up, modular assembly of lifeless components would be a major scientific breakthrough. However, to obtain a synthetic cell surviving throughout multiple generations, a certain level of homoeostasis of cell size and genomic content must be ensured. Disturbing the latter balance is associated with severe fitness-loss in natural cells^19,76^, and most likely also generates non-viable offspring of synthetic cells (Fig. 5a). We argue that the cell cycle of a synthetic cell should fulfil three minimal requirements. Firstly, replication must be coupled to cell growth, such that, independently from the chosen chromosomal configuration, the DNA density remains constant over many generations. Secondly, during DNA segregation, sister chromosomes must be correctly partitioned during the cell division process, such that each daughter cell contains at least one copy of the chromosome. Finally, cell division must be coordinated with growth in such a way that during each cell cycle a doubling in cell size occurs, thus achieving cell size homoeostasis over many generations (Box 1). In efforts to build a synthetic cell, we can explore and exploit the large variety of solutions that different natural life forms have evolved to couple these core processes.

Cell growth and the cell cycle are coordinated, yet separable processes. In most unicellular species, inhibiting cell growth causes a delay or even an arrest of the cell cycle^77,78^. Conversely, blocking or perturbing the cell cycle typically has a minor impact on cell growth^79,80^. While in some eukaryotes (e.g. fission yeast) a dependence of the growth rate on the stage of the cell cycle has been reported^81^, bacteria are known to grow exponentially at an approximately constant rate throughout the whole cell cycle^82,83^. Assuming that the minimal metabolism of a synthetic cell will probably lead to a continuous increase in cell volume (e.g. through lipid biosynthesis), the main challenge of controlling the cell cycle of a synthetic cell is to couple its core processes to growth and to coordinate the timing of the different events.

In most eukaryotes, the cell cycle is strictly regulated and consists of four discrete phases (Fig. 5b). An intricate system of molecular checkpoints and feedback control mechanisms leads to sequential activation of different transcriptional master regulators, which in turn trigger the transitions between different phases of the cell cycle^84^. A similar system is also present in the bacterium *Caulobacter crescentus*^85^. However, implementing even the simplest version of such a controlled cell cycle would be a considerable challenge, since it involves a large number of components and non-linear feedback loops^86,87^. For these reasons, simpler alternatives should be explored.

The cell cycle of the model bacterium *E. coli* appears to be less strictly regulated than its eukaryotic counterparts, as the different phases of the bacterial cell cycle are not strictly separated by checkpoints^88^ (Fig. 5c). Despite the lack of master regulators, *E. coli* cells exhibit a robust cell cycle that even allows them to divide faster than the time it takes to complete DNA replication^89,90^. This apparent paradox was resolved by early studies reporting that at high growth rates, *E. coli* cells start a new round of chromosomal replication before the previous one is finished, giving rise to chromosomes with multiple replication forks^91^. It was initially proposed that DNA replication is initiated in *E. coli* at a critical volume per origin of replication, independently of the growth rate^91^. Indeed, recent single-cell experiments provide strong experimental support for this prediction^90,92^. In addition, it was observed that, in total, the time required for the DNA replication and the cell division processes together is roughly constant. Overall, this led to the idea that replication initiation and cell division are co-regulated with growth^93^ via a combination of so-called “sizer” and “timer” mechanisms (Box 1). In this scenario, *E. coli* initiates chromosomal replication at a constant volume per origin (sizer) and divides a fixed time later (timer), yielding stable cell cycles.

Yet, recent studies have challenged this idea^92,94^. *E. coli* adds a constant volume each generation, independently from its size at birth, a concept known as “adder” (Box 1). At closer inspection, the “adder” principle is also valid for the added volume between replication initiation and cell division and even between replication initiation events of successive cell cycles, leading to “double adder” behaviour^92,94^. This suggests that DNA replication and cell division, while both individually coupled to growth, might be less coordinated with each other than previously thought.

It is commonly believed that DnaA and FtsZ^94^ are the central components that regulate bacterial DNA replication and cell division, respectively. The DnaA system controlling replication initiation in *E. coli* has been fairly well-characterised and could therefore be potentially used to control DNA replication by the aforementioned ϕ29 machinery (Fig. 2). Replication by the ϕ29 system starts at the origin of replication through helicase-based unwinding of the linear, double-stranded phage DNA, but this is an uncontrolled process that normally leads to a replication burst that is typical for phage proliferation. In a synthetic cell, control mechanisms could be designed to couple expression of the ϕ29 helicase to the cell cycle. Alternatively, substituting the uncontrolled viral helicase by the well-controlled bacterial DnaA system would allow a synthetic cell to exert strict control over the initiation step of chromosomal replication, while synthesis of the new DNA strands would be mediated by the minimal ϕ29 replicative machinery.

How DnaA and FtsZ control chromosome replication initiation and cell division is not yet completely understood. A long-standing idea has been that these processes are timed via protein accumulation up to a threshold level^95–97^. However, the initiator protein DnaA exists in two states, either active origin-unwinding (ATP-bound) or inactive (ADP-bound), and most likely this activation switch is responsible for the coupling of replication initiation to growth^88^. Despite extensive research^98^, however, a mechanistic understanding of this system is still lacking. Therefore, simplified synthetic solutions based on the accumulation of an initiator protein up to a threshold level could be considered for implementation in a synthetic cell.

Plasmid copy-number control systems provide alternative mechanisms for coordinating replication with growth. Plasmids are extrachromosomal genetic elements that, in some instances, replicate independently of the chromosome of the host^99,100^. Most, if not all, plasmids regulate the initiation of their replication based on their intracellular concentration in order to maintain a constant plasmid density^101–103^. Three mechanisms have been described for control of plasmid replication initiation, all mediated by different plasmid-encoded regulators: a negatively autoregulated transcription regulator protein^101,104^, inhibitory antisense RNAs^105^, or a plasmid-binding protein that inhibits replication through cross-linking plasmids at elevated concentrations (“handcuffing”)^103^. In addition to these replication control mechanisms, some plasmids have evolved addiction mechanisms, such as toxin/anti-toxin systems, to enhance the equal partitioning over daughter cells^106^. While plasmid copy-number control systems maintain a constant DNA density, they do not ensure simultaneous replication at a constant cell volume. The lack of coupling DNA replication initiation to cell division, appears to hamper the potential of these plasmid control systems as regulators in a synthetic cell cycle.

Box 1 Phenomenological models for cell size and DNA density control in bacteria

All organisms need to maintain a stable average cell size in a variety of different growth conditions. Symmetrically dividing cells must, on average, double their volume once per cell cycle, such that the average division volume 〈V_d_〉 is twice the average birth volume 〈V_b_〉. In the presence of biological noise (e.g. non-symmetrical cell division), cells can be born bigger or smaller than average (V_b_ = 〈V_b_〉 + δV). In order to maintain cell size homoeostasis, it is imperative to reduce deviations in birth size over the course of the cell cycle and over several generations. Panel (a) shows how the size at division, V_d_, depends on the size at birth, V_b_, in three different phenomenological models of cell size homoeostasis in exponentially growing cells. In the “sizer” model, the division volume is independent of the birth volume and equals twice the average birth volume (V_d_ = 2〈V_b_〉). Therefore, the “sizer” reduces noise in the birth volume within one generation only, providing the optimal division size control. In the “timer” model, cells divide a constant time after birth. In exponentially growing cells, the birth volume (V_b_) is doubled (2V_b_) after a constant time (τ_d_), set by the growth rate of the cell. Fluctuations in the birth volume are therefore not reduced upon cell division. In fact, in the entire unstable region shown in panel (a), cell size homoeostasis is not maintained, because initial variations in the birth volume are further amplified instead of reduced. Thus, the “timer” does not ensure a stable cell size. The “adder” behaviour, based on more recent single-cell measurements, reduces fluctuations in the birth volume by adding an on average constant volume 〈V_b_〉 from birth to division. An initial deviation in the birth volume δV is reduced by a factor of two in every generation, thus ensuring cell size homoeostasis over generations.

In addition to cell size control, cells must also maintain a constant chromosome density and couple DNA replication to cell division. It has been suggested that *E. coli* achieves this by initiating replication at a constant volume per number of origins of replication, and dividing an approximately constant time later (panel (b)). This combination of a sizer for DNA replication and a timer for cell division reduces fluctuations in the birth volume on the level of replication initiation and a stable cell size is maintained at all growth conditions. Additionally, this simple mechanism ensures that every replication initiation event is followed by a cell division, thus also coupling replication to division.

The biochemical networks regulating the timing of replication initiation and cell division are often very complex. Until today, it remains poorly understood what molecular mechanisms give rise to the phenomenological observations, even in well-studied bacteria such as *E. coli*. These phenomenological models, however, provide a good framework for designing regulation modules that ensure a robust cell cycle of a synthetic cell.

## Integration and compatibility of a synthetic cell cycle

**Fig. 6:**
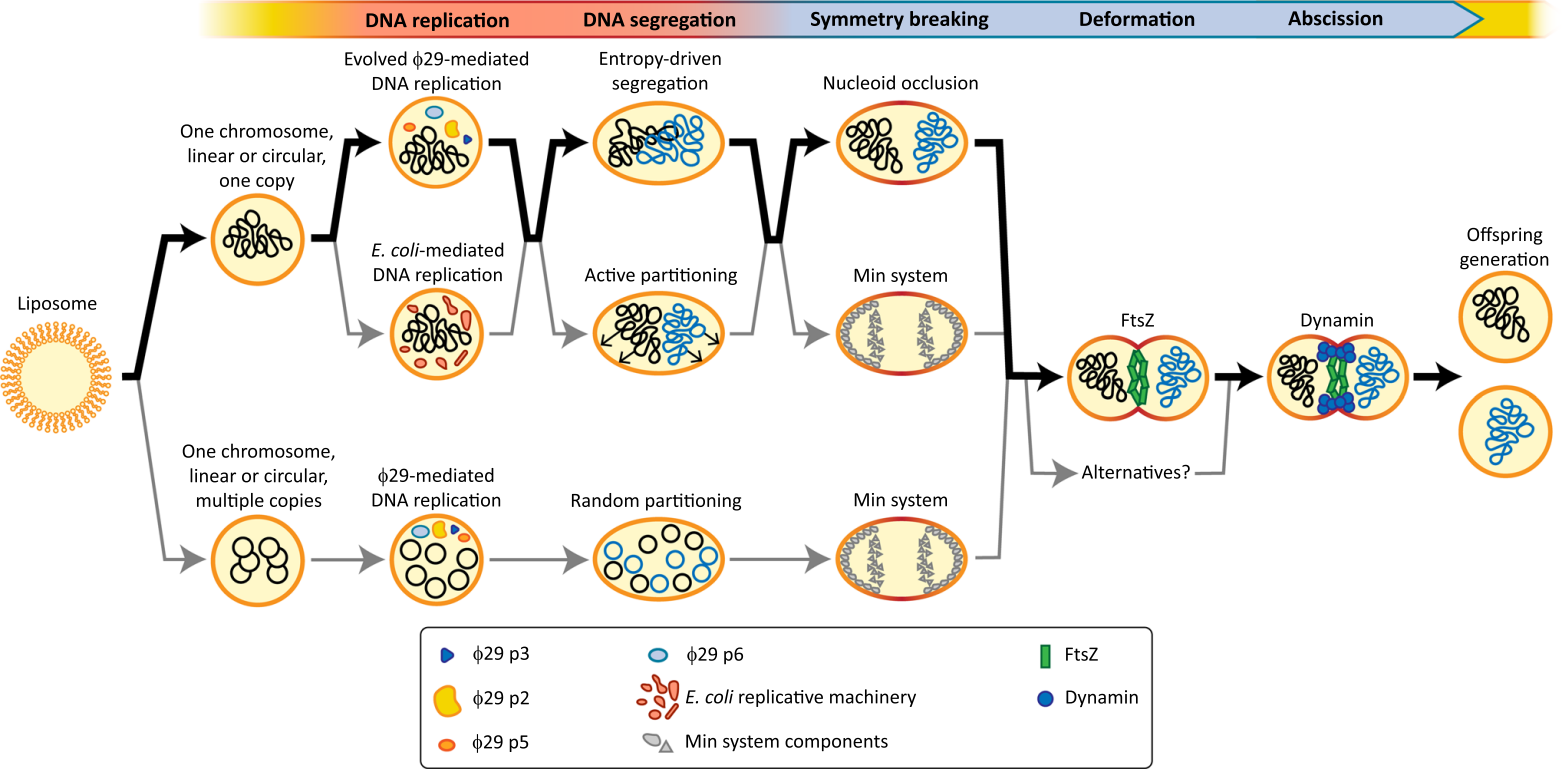
Towards a synthetic cell cycle. Various possible routes are indicated to accomplish a sustainable synthetic cell cycle. The pathway that, in our opinion, is most promising based on current insights, is marked in bold black arrows. Alternative paths are depicted in thin grey arrows. Note that not all possible alternative paths are displayed and that we only depicted circular chromosomes here. However, a linear chromosome architecture could also be a viable option, especially in combination with the φ29 machinery.

To conclude this perspective, we will discuss a few potential routes towards building a synthetic cell cycle (Fig. 6). The presented routes have been selected on basis of the aforementioned insights of individual module variants, as well as on considerations on their functional compatibility. First, the presence of a container is essential to define the context of the synthetic cell. Being at the centre of extensive research throughout the years^107^, inverted emulsion-generated liposomes of a defined size (1–5 μm diameter), and composed of selected bacterial lipids are considered the best choice to fulfil this role.

As to the chromosome configuration, we propose to include all genetic information on a single, circular or linear dsDNA chromosome (total 250–500 kb). Because of its simplicity and its reported in vitro performance^14,17^, the ϕ29 system appears very attractive for DNA replication, although, as discussed, its processivity and its preference for linear chromosomes may require optimisation to cope with replication of chromosomes up to 500 kb. In addition, implementing a sizer-like modulation of replication activity (Box 1), would require including an additional control module, for which the natural *E. coli* DnaA-mediated regulation of replication initiation could serve as inspiration.

Another important design criterium to be considered concerns the copy number of the chromosome. Employing multiple copies of the chromosome means that a plasmid-like copy number control mechanism, together with random partitioning, could be sufficient for dividing the chromosomes over the daughter cells. However, as simultaneous initiation of replication of all the copies at a certain volume could not be easily controlled in this scenario, division cannot be coupled to replication via a timer. Thus, an additional control module should be implemented to ensure cell size homoeostasis through generations. Another important consequence of multiple chromosomes would be that symmetry breaking cannot operate through nucleoid occlusion. Hence, a more appealing alternative is to only use a single copy of the chromosome, and to ensure a sufficient degree of confinement by generating liposomes of appropriate dimensions. Importantly, this genomic configuration would allow for employing entropy-driven segregation. Compared to alternatives, this mechanism would considerably reduce the molecular complexity of the synthetic cell. If entropy-driven chromosome segregation alone proves to be insufficient to break the cell symmetry, implementation of the Min system could be considered.

As nucleoid occlusion prevents assembly of membrane-deforming machineries close to the chromosome^52^, the cell division process appears to initiate only after full partitioning occurred, thus achieving an intrinsic timer behaviour in synthetic cells that employ a sufficiently confined chromosome. Subsequently, FtsZ can assemble the Z-ring at the cell midpoint to induce membrane deformation. FtsZ is to be initially preferred over other systems due to the natural connection with other well-known constriction and abscission mechanisms, although it still remains to be confirmed whether the Z-ring alone is able to constrict a membrane. Finally, bacterial dynamin can be recruited at the Z-ring and promote membrane fission to complete the cell division. This process then may be expected to generate two daughter cells, ready to undergo a new round of the synthetic cell cycle. Integrating a sizer for replication initiation and a timer for division is considered a promising combination that might give rise to robust cell cycles in a synthetic cell.

Apart from the modular routes proposed here (Fig. 6), several alternative sets of modules could be proposed. Despite individual preferences, we believe that the interconnected nature of the processes taking part in the cell cycle should be considered as a key feature when developing a synthetic cell. Smart design and tuning by evolution eventually should provide a robust and well-integrated synthetic cell cycle that will be a major step towards a synthetic cell that grows and divides autonomously.

## References

[1] Hutchison, C. A. et al. Design and synthesis of a minimal bacterial genome. *Science* **351**, 10.1126/science.aad6253 (2016). 10.1126/science.aad6253 27013737

[2] Pelletier, J. F. et al. Genetic requirements for cell division in a genomically minimal cell. *Cell*, 10.1016/j.cell.2021.03.008 (2021). 10.1016/j.cell.2021.03.008 33784496

[3] Sikkema HR, Gaastra BF, Pols T, Poolman B (2019). Cell fuelling and metabolic energy conservation in synthetic cells. ChemBioChem.

[4] Gaut, N. J. & Adamala, K. P. Reconstituting natural cell elements in synthetic cells. *Adv. Biol*., 10.1002/adbi.202000188 (2021). 10.1002/adbi.202000188 33729692

[5] Abil, Z. & Danelon, C. Roadmap to building a cell: an evolutionary approach. *Front. Bioeng. Biotechnol*. **8**, 10.3389/fbioe.2020.00927 (2020). 10.3389/fbioe.2020.00927 PMC7466671 32974299

[6] Forster, A. C. & Church, G. M. Towards synthesis of a minimal cell. *Mol. Syst. Biol.* **2**, 10.1038/msb4100090 (2006). 10.1038/msb4100090 PMC1681520 16924266

[7] Fujiwara K, Katayama T, Nomura SIM (2013). Cooperative working of bacterial chromosome replication proteins generated by a reconstituted protein expression system. Nucleic Acids Res..

[8] Su’etsugu M, Takada H, Katayama T, Tsujimoto H (2017). Exponential propagation of large circular DNA by reconstitution of a chromosome-replication cycle. Nucleic Acids Res..

[9] Schaerli Y (2010). Isothermal DNA amplification using the T4 replisome: circular nicking endonuclease-dependent amplification and primase-based whole-genome amplification. Nucleic Acids Res..

[10] Hürtgen D (2019). Reconstitution and coupling of DNA replication and segregation in a biomimetic system. ChemBioChem.

[11] Salas, M., Holguera, I., Redrejo-Rodríguez, M. & Vega, M. de. DNA-binding proteins essential for protein-primed bacteriophage ϕ29 DNA replication. *Front. Mol. Biosci.* **3**, 10.3389/fmolb.2016.00037 (2016). 10.3389/fmolb.2016.00037 PMC4974454 27547754

[12] Paez, J. G. et al. Genome coverage and sequence fidelity of phi29 polymerase-based multiple strand displacement whole genome amplification. *Nucleic Acids Res*. **32**, 10.1093/nar/gnh069 (2004). 10.1093/nar/gnh069 PMC419624 15150323

[13] Blanco L (1989). Highly efficient DNA synthesis by the phage ϕ29 DNA polymerase. Symmetrical mode of DNA replication. J. Biol. Chem..

[14] Van Nies, P. et al. Self-replication of DNA by its encoded proteins in liposome-based synthetic cells. *Nat. Commun*. **9**, 10.1038/s41467-018-03926-1 (2018). 10.1038/s41467-018-03926-1 PMC5910420 29679002

[15] Sakatani, Y., Ichihashi, N., Kazuta, Y. & Yomo, T. A transcription and translation-coupled DNA replication system using rolling-circle replication. *Sci. Rep*. **5**, 10.1038/srep10404 (2015). 10.1038/srep10404 PMC4445062 26013404

[16] Sakatani, Y., Yomo, T. & Ichihashi, N. Self-replication of circular DNA by a self-encoded DNA polymerase through rolling-circle replication and recombination. *Sci. Rep*. **8**, 10.1038/s41598-018-31585-1 (2018). 10.1038/s41598-018-31585-1 PMC6117322 30166584

[17] Libicher, K., Hornberger, R., Heymann, M. & Mutschler, H. In vitro self-replication and multicistronic expression of large synthetic genomes. *Nat. Commun*. **11**, 10.1038/s41467-020-14694-2 (2020). 10.1038/s41467-020-14694-2 PMC7021806 32060271

[18] Sakatani Y, Mizuuchi R, Ichihashi N (2019). In vitro evolution of phi29 DNA polymerases through compartmentalized gene expression and rolling-circle replication. Protein Eng. Des. Sel..

[19] Simmons LA, Breier AM, Cozzarelli NR, Kaguni JM (2004). Hyperinitiation of DNA replication in *Escherichia coli* leads to replication fork collapse and inviability. Mol. Microbiol..

[20] Bouck DC, Joglekar AP, Bloom KS (2008). Design features of a mitotic spindle: balancing tension and compression at a single microtubule kinetochore interface in budding yeast. Annu. Rev. Genet..

[21] Gerdes K, Howard M, Szardenings F (2010). Pushing and pulling in prokaryotic DNA segregation. Cell.

[22] Nordstrom K, Austin SJ (1989). Mechanisms that contribute to the stable segregation of plasmids. Annu. Rev. Genet..

[23] Huh D, Paulsson J (2011). Random partitioning of molecules at cell division. Proc. Natl Acad. Sci..

[24] Vleugel, M., Roth, S., Groenendijk, C. F. & Dogterom, M. Reconstitution of Basic Mitotic Spindles in Spherical Emulsion Droplets. *J. Vis. Exp*. **114**, 10.3791/54278 (2016). 10.3791/54278 PMC5091848 27584979

[25] Ietswaart, R., Szardenings, F., Gerdes, K. & Howard, M. Competing ParA Structures Space Bacterial Plasmids Equally over the Nucleoid. *PLoS Comput. Biol*. **10**, 10.1371/journal.pcbi.1004009 (2014). 10.1371/journal.pcbi.1004009 PMC4270457 25521716

[26] Woldringh CL (2002). The role of co-transcriptional translation and protein translocation (transertion) in bacterial chromosome segregation. Mol. Microbiol..

[27] Woldringh, C. L., Hansen, F. G., Vischer, N. O. E. & Atlung, T. Segregation of chromosome arms in growing and non-growing *Escherichia coli* cells. *Front. Microbiol*. **6**, 10.3389/fmicb.2015.00448 (2015). 10.3389/fmicb.2015.00448 PMC4428220 26029188

[28] Lemon KP (2001). The extrusion-capture model for chromosome partitioning in bacteria. Genes Dev..

[29] Rocha EPC (2002). Is there a role for replication fork asymmetry in the distribution of genes in bacterial genomes?. Trends Microbiol..

[30] Gitai Z, Thanbichler M, Shapiro L (2005). The choreographed dynamics of bacterial chromosomes. Trends Microbiol..

[31] Jun S, Mulder B (2006). Entropy-driven spatial organization of highly confined polymers: lessons for the bacterial chromosome. Proc. Natl Acad. Sci..

[32] De Gennes, P. G. *Scaling Concepts in Polymer Physics*. *Physics Today* **33**, 6, 51 (1980).

[33] Jun S, Wright A (2010). Entropy as the driver of chromosome segregation. Nat. Rev. Microbiol..

[34] Ha B-Y, Jung Y (2015). Polymers under confinement: single polymers, how they interact, and as model chromosomes. Soft Matter.

[35] Minina E, Arnold A (2015). Entropic Segregation of Ring Polymers in Cylindrical Confinement. Macromolecules.

[36] Bohn, M. & Heermann, D. W. Topological interactions between ring polymers: Implications for chromatin loops. *J. Chem. Phys*. **132**, 10.1063/1.3302812 (2010). 10.1063/1.3302812 20113063

[37] Krogh, T. J., Møller-Jensen, J. & Kaleta, C. Impact of chromosomal architecture on the function and evolution of bacterial genomes. *Front. Microbiol*. **9**,10.3389/fmicb.2018.02019 (2018). 10.3389/fmicb.2018.02019 PMC6119826 30210483

[38] Shin J, Cherstvy AG, Metzler R (2014). Mixing and segregation of ring polymers: spatial confinement and molecular crowding effects. N. J. Phys..

[39] Japaridze, A., Gogou, C., Kerssemakers, J. W. J., Nguyen, H. M. & Dekker, C. Direct observation of independently moving replisomes in *Escherichia coli*. *Nat. Commun*. **11**, 10.1038/s41467-020-16946-7 (2020). This paper indicates that cell boundary confinement and entropy are important drivers of DNA segregation. 10.1038/s41467-020-16946-7 PMC7305307 32561741

[40] Wu, L. J. et al. Geometric principles underlying the proliferation of a model cell system. *Nat. Commun*. **11**, 10.1038/s41467-020-17988-7 (2020). 10.1038/s41467-020-17988-7 PMC7434903 32811832

[41] Zubaite, G. et al. Droplet Microfluidics Approach for Single-DNA Molecule Amplification and Condensation into DNA-Magnesium-Pyrophosphate Particles. *Micromachines* **8**, 10.3390/mi8020062 (2017).

[42] Drobot, B. et al. Compartmentalised RNA catalysis in membrane-free coacervate protocells. *Nat. Commun*. **9**, 10.1038/s41467-018-06072-w (2018). 10.1038/s41467-018-06072-w PMC6128941 30194374

[43] Litschel T, Ramm B, Maas R, Heymann M, Schwille P (2018). Beating vesicles: encapsulated protein oscillations cause dynamic membrane deformations. Angew. Chem. Int. Ed..

[44] Godino, E. et al. De novo synthesized Min proteins drive oscillatory liposome deformation and regulate FtsA-FtsZ cytoskeletal patterns. *Nat. Commun*. **10**, 10.1038/s41467-019-12932-w (2019). 10.1038/s41467-019-12932-w PMC6823393 31672986

[45] Garten, M., Aimon, S., Bassereau, P. & Toombes, G. E. S. Reconstitution of a transmembrane protein, the voltage-gated ion channel, KvAP, into giant unilamellar vesicles for microscopy and patch clamp studies. *J. Vis. Exp*. **95**, 10.3791/52281 (2015). 10.3791/52281 PMC4354550 25650630

[46] Ramm B, Heermann T, Schwille P (2019). The *E. coli* MinCDE system in the regulation of protein patterns and gradients. Cell. Mol. Life Sci..

[47] Ramm, B. et al. The MinDE system is a generic spatial cue for membrane protein distribution in vitro. *Nat. Commun*. **9**, 10.1038/s41467-018-06310-1 (2018). 10.1038/s41467-018-06310-1 PMC6158289 30258191

[48] Nance J, Zallen JA (2011). Elaborating polarity: PAR proteins and the cytoskeleton. Development.

[49] Vendel, K. J. A., Tschirpke, S., Shamsi, F., Dogterom, M. & Laan, L. Minimal in vitro systems shed light on cell polarity. *J. Cell Sci*. **132**, 10.1242/jcs.217554 (2019). 10.1242/jcs.217554 30700498

[50] Padirac A, Fujii T, Estévez-Torres A, Rondelez Y (2013). Spatial waves in synthetic biochemical networks. J. Am. Chem. Soc..

[51] Shimobayashi SF, Ichikawa M, Taniguchi T (2016). Direct observations of transition dynamics from macro- to micro-phase separation in asymmetric lipid bilayers induced by externally added glycolipids. EPL.

[52] Wu LJ, Errington J (2012). Nucleoid occlusion and bacterial cell division. Nat. Rev. Microbiol..

[53] Caspi Y, Dekker C (2014). Divided we stand: splitting synthetic cells for their proliferation. Syst. Synth. Biol..

[54] Dreher, Y., Jahnke, K., Bobkova, E., Spatz, J. P. & Göpfrich, K. Division and Regrowth of Phase-Separated Giant Unilamellar Vesicles. *Angew. Chemie - Int. Ed.* **60**, 10661–10669 (2021). 10.1002/anie.202014174 PMC8252472 33355974

[55] Ganzinger KA (2020). FtsZ reorganization facilitates deformation of giant vesicles in microfluidic traps. *Angew*. Chem. Int. Ed..

[56] Ramirez-Diaz, D.A., Merino-Salomón, A., Meyer, F. *et al.* FtsZ induces membrane deformations via torsional stress upon GTP hydrolysis. *Nat Commun* **12**, 3310 (2021). 10.1038/s41467-021-23387-3 PMC8175707 34083531

[57] Godino, E. et al. Cell-free biogenesis of bacterial division proto-rings that can constrict liposomes. *Commun. Biol*. **3**, 10.1038/s42003-020-01258-9 (2020). 10.1038/s42003-020-01258-9 PMC7527988 32999429

[58] Zieske, K. & Schwille, P. Reconstitution of self-organizing protein gradients as spatial cues in cell-free systems. *Elife* **3**, 10.7554/eLife.03949 (2014). 10.7554/eLife.03949 PMC4215534 25271375

[59] Furusato T (2018). De Novo Synthesis of Basal Bacterial Cell Division Proteins FtsZ, FtsA, and ZipA Inside Giant Vesicles. ACS Synth. Biol..

[60] Cabré EJ (2013). Bacterial division proteins FtsZ and ZipA induce vesicle shrinkage and cell membrane invagination. J. Biol. Chem..

[61] Lee KY (2018). Photosynthetic artificial organelles sustain and control ATP-dependent reactions in a protocellular system. Nat. Biotechnol..

[62] Loiseau, E. *et al*. Shape remodeling and blebbing of active cytoskeletal vesicles. *Sci. Adv*. **2**, 10.1126/sciadv.1500465 (2016). 10.1126/sciadv.1500465 PMC4846454 27152328

[63] Tsai F-C, Koenderink GH (2015). Shape control of lipid bilayer membranes by confined actin bundles. Soft Matter.

[64] Tanaka, S., Takiguchi, K. & Hayashi, M. Repetitive stretching of giant liposomes utilizing the nematic alignment of confined actin. *Commun. Phys*. **1**, 10.1038/s42005-018-0019-2 (2018).

[65] Hayashi M (2016). Reversible morphological control of tubulin-encapsulating giant liposomes by hydrostatic pressure. Langmuir.

[66] Keber FC (2014). Topology and dynamics of active nematic vesicles. Science.

[67] Sato, Y., Hiratsuka, Y., Kawamata, I., Murata, S. & Nomura, S. M. Micrometer-sized molecular robot changes its shape in response to signal molecules. *Sci. Robot*. **2**, 10.1126/scirobotics.aal3735 (2017). 10.1126/scirobotics.aal3735 33157867

[68] Tarrason Risa, G. *et al.* The proteasome controls ESCRT-III-mediated cell division in an archaeon. *Science* **369**, (2020). 10.1126/science.aaz2532 PMC7116001 32764038

[69] Ramezani H, Dietz H (2019). Building machines with DNA molecules. Nat. Rev. Genet..

[70] Franquelim, H. G., Khmelinskaia, A., Sobczak, J.-P., Dietz, H. & Schwille, P. Membrane sculpting by curved DNA origami scaffolds. *Nat. Commun*. **9**, 10.1038/s41467-018-03198-9 (2018). 10.1038/s41467-018-03198-9 PMC5824810 29476101

[71] Grome MW, Zhang Z, Lin C (2019). Stiffness and membrane anchor density modulate DNA-nanospring-induced vesicle tubulation. ACS Appl. Mater. Interfaces.

[72] Schöneberg J (2018). ATP-dependent force generation and membrane scission by ESCRT-III and Vps4. Science.

[73] Schlimpert S (2017). Two dynamin-like proteins stabilize FtsZ rings during *Streptomyces* sporulation. Proc. Natl Acad. Sci..

[74] Weirich KL, Dasbiswas K, Witten TA, Vaikuntanathan S, Gardel ML (2018). Self-organizing motors divide active liquid droplets. Proc. Natl Acad. Sci..

[75] Last MGF, Deshpande S, Dekker C (2020). pH-controlled coacervate-membrane interactions within liposomes. ACS Nano.

[76] Fernandez-Fernandez C, Gonzalez D, Collier J (2011). Regulation of the activity of the dual-function DnaA protein in *Caulobacter crescentus*. PLoS One.

[77] Johnston GC, Pringle JR, Hartwell LH (1977). Coordination of growth with cell division in the yeast *Saccharomyces cerevisiae*. Exp. Cell Res..

[78] Ferullo DJ, Lovett ST (2008). The stringent response and cell cycle arrest in *Escherichia coli*. PLoS Genet..

[79] Jorgensen P, Tyers M (2004). How cells coordinate growth and division. Curr. Biol..

[80] Wehrens M (2018). Size laws and division ring dynamics in filamentous *Escherichia coli* cells. Curr. Biol..

[81] Pickering M, Hollis LN, D’Souza E, Rhind N (2019). Fission yeast cells grow approximately exponentially. Cell Cycle.

[82] Iyer-Biswas S (2014). Scaling laws governing stochastic growth and division of single bacterial cells. Proc. Natl Acad. Sci. USA..

[83] Soifer I, Robert L, Amir A (2016). Single-cell analysis of growth in budding yeast and bacteria reveals a common size regulation strategy. Curr. Biol..

[84] Cooper, G. M. The Eukaryotic Cell Cycle. in *The Cell: A Molecular Approach*. *2nd edition* (Sinauer Associates, Inc., 2000).

[85] Skerker JM, Laub MT (2004). Cell-cycle progression and the generation of asymmetry in *Caulobacter crescentus*. Nat. Rev. Microbiol..

[86] Tanaka S (2007). CDK-dependent phosphorylation of Sld2 and Sld3 initiates DNA replication in budding yeast. Nature.

[87] Kraikivski, P., Chen, K. C., Laomettachit, T., Murali, T. M. & Tyson, J. J. From START to FINISH: Computational analysis of cell cycle control in budding yeast. *npj Syst. Biol. Appl*. **1**, 10.1038/npjsba.2015.16 (2015). 10.1038/npjsba.2015.16 PMC5516803 28725464

[88] Reyes-Lamothe R, Sherratt DJ (2019). The bacterial cell cycle, chromosome inheritance and cell growth. Nat. Rev. Microbiol..

[89] Cooper S, Helmstetter CE (1968). Chromosome replication and the division cycle of *Escherichia coli* B/r. J. Mol. Biol..

[90] Wallden M, Fange D, Lundius EG, Baltekin Ö, Elf J (2016). The synchronization of replication and division cycles in individual *E. coli*. Cells Cell.

[91] Donachie WD (1968). Relationship between cell size and time of initiation of DNA replication. Nature.

[92] Witz, G., Van Nimwegen, E. & Julou, T. Initiation of chromosome replication controls both division and replication cycles in *E. coli* though a double-adder mechanism. *Elife* **8**, 10.7554/eLife.48063 (2019). 10.7554/eLife.48063 PMC6890467 31710292

[93] Si F (2017). Invariance of initiation mass and predictability of cell size in *Escherichia coli*. Curr. Biol..

[94] Si F (2019). Mechanistic origin of cell-size control and homeostasis in bacteria. Curr. Biol..

[95] Somoayrac L, Maaloe O (1973). Autorepressor model for control of DNA replication. Nat. N. Biol..

[96] Ho, P.-Y. & Amir, A. Simultaneous Regulation of Cell Size and Chromosome Replication in Bacteria. *Front. Microbiol*. **6**, 10.3389/fmicb.2015.00662 (2015). 10.3389/fmicb.2015.00662 PMC4498127 26217311

[97] Basan, M. *et al*. Inflating bacterial cells by increased protein synthesis. *Mol. Syst. Biol*. **11**, 10.15252/msb.20156178 (2015). 10.15252/msb.20156178 PMC4631207 26519362

[98] Katayama, T., Kasho, K. & Kawakami, H. The DnaA cycle in *Escherichia coli*: activation, function and inactivation of the initiator protein. *Front. Microbiol.* **8**, 10.3389/fmicb.2017.02496 (2017). 10.3389/fmicb.2017.02496 PMC5742627 29312202

[99] Chattoraj DK, Mason RJ, Wickner SH (1988). Mini-P1 plasmid replication: the autoregulation-sequestration paradox. Cell.

[100] Egan ES, Løbner-Olesen A, Waldor MK (2004). Synchronous replication initiation of the two *Vibrio cholerae* chromosomes. Curr. Biol..

[101] Lee SB, Bailey JE (1984). A mathematical model for λ dv plasmid replication: analysis of wild-type plasmid. Plasmid.

[102] Paulsson, J. & Ehrenberg, M. Noise in a minimal regulatory network: Plasmid copy number control. *Quarterly Rev. Biophys.* **34**, 10.1017/s0033583501003663 (2001). 10.1017/s0033583501003663 11388089

[103] Das N (2005). Multiple homeostatic mechanisms in the control of P1 plasmid replication. Proc. Natl Acad. Sci. USA..

[104] Nordström K (1990). Control of plasmid replication-How do DNA iterons set the replication frequency?. Cell.

[105] Paulsson J, Nordström K, Ehrenberg M (1998). Requirements for rapid plasmid ColE1 copy number adjustments: a mathematical model of inhibition modes and RNA turnover rates. Plasmid.

[106] Harms A, Brodersen DE, Mitarai N, Gerdes K (2018). Toxins, targets, and triggers: an overview of toxin-antitoxin biology. Mol. Cell.

[107] Rideau E, Dimova R, Schwille P, Wurm FR, Landfester K (2018). Liposomes and polymersomes: a comparative review towards cell mimicking. Chem. Soc. Rev..

[108] Deshpande S, Spoelstra WK, van Doorn M, Kerssemakers J, Dekker C (2018). Mechanical division of cell-sized liposomes. ACS Nano.

[109] Franquelim HG, Dietz H, Schwille P (2020). Reversible membrane deformations by straight DNA origami filaments. Soft Matter.

[110] Vutukuri HR (2020). Active particles induce large shape deformations in giant lipid vesicles. Nature.

[111] Garner EC, Campbell CS, Weibel DB, Mullins RD (2007). Reconstitution of DNA segregation driven by assembly of a prokaryotic actin homolog. Science.

[112] Wu H, Brennwald P (2010). The function of two Rho family GTPases is determined by distinct patterns of cell surface localization. Mol. Cell. Biol..

[113] Pols, T. *et al*. A synthetic metabolic network for physicochemical homeostasis. *Nat. Commun*. **10**, 10.1038/s41467-019-12287-2 (2019). 10.1038/s41467-019-12287-2 PMC6751199 31534136

[114] Xu C, Hu S, Chen X (2016). Artificial cells: from basic science to applications. Mater. Today.

[115] Blanken, D., Foschepoth, D., Serrão, A. C. & Danelon, C. Genetically controlled membrane synthesis in liposomes. *Nat. Commun*. **11**, 10.1038/s41467-020-17863-5 (2020). 10.1038/s41467-020-17863-5 PMC7455746 32859896

[116] Bhattacharya, A., Brea, R. J., Niederholtmeyer, H. & Devaraj, N. K. A minimal biochemical route towards de novo formation of synthetic phospholipid membranes. *Nat. Commun*. **10**, 10.1038/s41467-018-08174-x (2019). 10.1038/s41467-018-08174-x PMC6336818 30655537

[117] Kuruma Y, Stano P, Ueda T, Luisi PL (2009). A synthetic biology approach to the construction of membrane proteins in semi-synthetic minimal cells. Biochim. Biophys. Acta. Biomembr..

